# Humanized Care From the Nurse–Patient Perspective in a Hospital Setting: A Systematic Review of Experiences Disclosed in Spanish and Portuguese Scientific Articles

**DOI:** 10.3389/fpubh.2021.737506

**Published:** 2021-12-03

**Authors:** Monica Elisa Meneses-La-Riva, Josefina Amanda Suyo-Vega, Víctor Hugo Fernández-Bedoya

**Affiliations:** ^1^School of Nursing, Universidad César Vallejo, Lima, Peru; ^2^School of Education, Universidad César Vallejo, Lima, Peru; ^3^School of Management, Universidad César Vallejo, Lima, Peru

**Keywords:** humanized care, nurse, patient, perspective, hospital, systematic review

## Abstract

Nowadays, humanized care is an essential component in the field of health because the professional work of nursing seeks to provide quality services to patients who are suffering and fear illness or the dying process. Nurses recognize the need to incorporate humanized care into their daily work, as supported by Jean Watson, who states that caring entails establishing an adequate nurse–patient therapeutic relationship, where health education is a tool that promotes self-care in the patient, family, and community. The main objective of this work was to find scientific evidence on humanized care from the perspectives of nurses and hospitalized patients. To meet those research objectives, an exploratory systematic review of articles published in high-quality scientific journals from 2016 to 2020 using the PRISMA methodology in the Scopus and Scielo databases was conducted, yielding 26 studies that were analyzed. The findings show that nurses and patients perceive the need to remove the barriers that limit the advancement of humanized care in hospital institutions because they urgently demand that health professionals in all settings, especially critical ones, strengthen their humanizing role by sharing cordial, empathetic health experiences, and respecting their customs and beliefs during the hospitalization process. As a conclusion of the findings, the nurse–patient professionals agree that health personnel training is critical to providing humanized attention with quality in the hospital context, emphasizing that professional training should develop in practice soft skills, communication, safety environment, and human values.

## Introduction

The humanization of care is an essential element to achieve the promotion of well-being during the processes of care within health systems because the hospital environment is immersed in an imbalance of emotions resulting from the suffering and anxiety endured by the sick person and the family (1, 2).

Promoting humanized care during the daily work of health professionals implies promoting the universality of the right to health as an essential element and where there should be no distinction of any kind (3). This should be developed as an integral formation of every nursing student in the university classrooms (4, 5).

The humanization of health services seeks to raise the quality of care at the administrative and assistance levels, for comprehensive care and without financial difficulties throughout the life of the patient (6–8).

The essence of nursing care is founded on Jean Watson's (9–12) theory, which states that care necessitates a way of being able to interact and connect with the patient and the family, with the goal of providing an environment of excellence, comfort, and safety, with high competencies, skills, and conditions to achieve holistic care (13).

Some authors agree that the conceptualization of care, the essential art of nursing, is to care for the most primitive act that a human being performs to effectively become, this being, a being in relation to another who invokes it in an ethical and philosophical foundation (14–19). Humanized care represents an enlightening guide and support to the practice for its benefit in cultivating a conscience of care and the establishment of a strong and sustainable therapeutic relationship. Therefore, care represents the work of nursing, based on humanistic values in order to meet the needs of patients and improve their quality of life (20, 21).

Care, as the essence of professional nursing practice, necessitates that professionals know how to act with ethical commitment, foster relationships of understanding, empathy, and respect, and prioritize actions to provide timely responses to difficulties, because humanized care, to be meaningful, must be based on the reciprocity of professionals in the face of health problems (22–24). However, no one is unaware that health services are seen as stressful and overcrowded places for patients, which causes emotional exhaustion in professionals as a result of work overload and lack of resources, which makes care management a current challenge for health professionals, especially for nurses (25, 26).

On the other hand, the dynamics of action of nursing professionals is to provide comprehensive care from the practice of learned scientific knowledge and their own experience, with the objective that the patient achieves physical, psychosocial, and spiritual well-being (27); since care has ethical bases, originates autonomy, and creates confidence and security (28).

In reference to the problematic reality, it can still be seen that nurses still govern their work in a technical–scientific and somewhat mechanized manner, often disregarding the human aspect, which alienates the patient and damages interpersonal relationships (29). In this regard, the humanized care approach conceives that “the other human being” in front of us, expects humanized care, being necessary that professionals have the appropriate qualities, which should be generated during academic training (30).

There are some studies that sought to systematize the evidence of the perception of humanized care received by patients or delivered by nurses throughout the world. Some cases can be reviewed in (31–33).

A systematic review was carried out in Chile on humanized care in patients with limited therapeutic effort focused on adults in intensive care in Ibero-America. The authors found 23 articles from the databases PubMed, Epistemonikos, Web of Science, Scielo, Elsevier, and added information from other electronic sources such as End-of-Life Journal and Journal of Medical Ethics. The main conclusion of the study was the determination that the nursing professional is aware of the diversity of roles he or she fulfills when attending to a patient, but when applying humanized care, the relationship between patient, family, and medical team is evidenced, communication being a priority agent for the knowledge of the needs of the patient (34).

Likewise, a systematic review on humanized care in children was carried out in Italy, analyzing scientific evidence published in the PubMed and Scopus databases. Only 28 records met the inclusion criteria, and it was concluded that humanized care is elementary for pediatric hospital global management, being necessary to continue research to strengthen child-centered research (31).

In Spain, the results of a systematic review on the humanized care of the neonate and the family were disclosed. The search was done in PubMed, Cochrane, CINHAL, Scopus, and Google Scholar databases. Thirteen peer-reviewed scientific articles were found that met the inclusion criteria, whose conclusions suggest the promotion of family participation through the modification of health policies such as the daily and permanent attention of the nurse and their constant communication (33).

Currently, health institutions show an urgent need to strengthen the right and guarantee accessibility to health services with a human face. Thus, humanized care is a way to raise the quality of health services provided by health professionals, especially nurses, who must create a safe environment, with dignified treatment for the patient, family, and community. The satisfaction experienced by patients and families in health institutions is important to strengthen trust, quality of services in a safe environment to ensure holistic care in each individual, taking into account a moral commitment, through values of respect for the dignity of life, contributing to improve the quality of life of people (13).

To complement the findings identified by the authors previously developed, it was deemed convenient to carry out a systematic review on humanized care in hospitalized patients, compiling evidence published in Spanish in the Scopus and Scielo databases.

## Materials and Methods

The study was an exploratory systematic review (35). We searched for evidence seeking to meet the research objectives in the Scopus and Scielo databases published in the years 2016–2020 in Spanish and Portuguese. The searches detailed in Table 1 were performed.

**Table 1:** Searches.

**Code**	**Database**	**Search**	**Language**
A1	Scopus	“Cuidado humanizado” AND “hospitalización”	Spanish
A2	Scopus	“Cuidados humanizados” AND “hospitalização”	Portuguese
B1	Scopus	“Paciente hospitalizado” AND “hospitalización”	Spanish
B2	Scopus	“Paciente hospitalizado” AND “Hospitalização”	Portuguese
C1	Scielo	“Cuidado humanizado” AND “hospitalización”	Spanish
C2	Scielo	“Cuidados humanizados” AND “hospitalização”	Portuguese
D1	Scielo	“Paciente hospitalizado” AND “hospitalización”	Spanish
D2	Scielo	“Paciente hospitalizado” AND “Hospitalização”	Portuguese

The initial search of the Scopus and Scielo databases yielded 74 records. During their exploration, 32 articles were identified that were neither downloadable nor visible, which were withdrawn from the study, resulting in a subtotal of 42 accessible records. The application of filters according to the inclusion and exclusion criteria resulted in 30 eligible records, of which four records were removed due to duplicity. The final result included 26 records. Having all the information collected, we proceeded according to the research question: What is the scientific evidence on humanized care from the point of view of nurse–patients in a hospital context?

**Figure 1:**
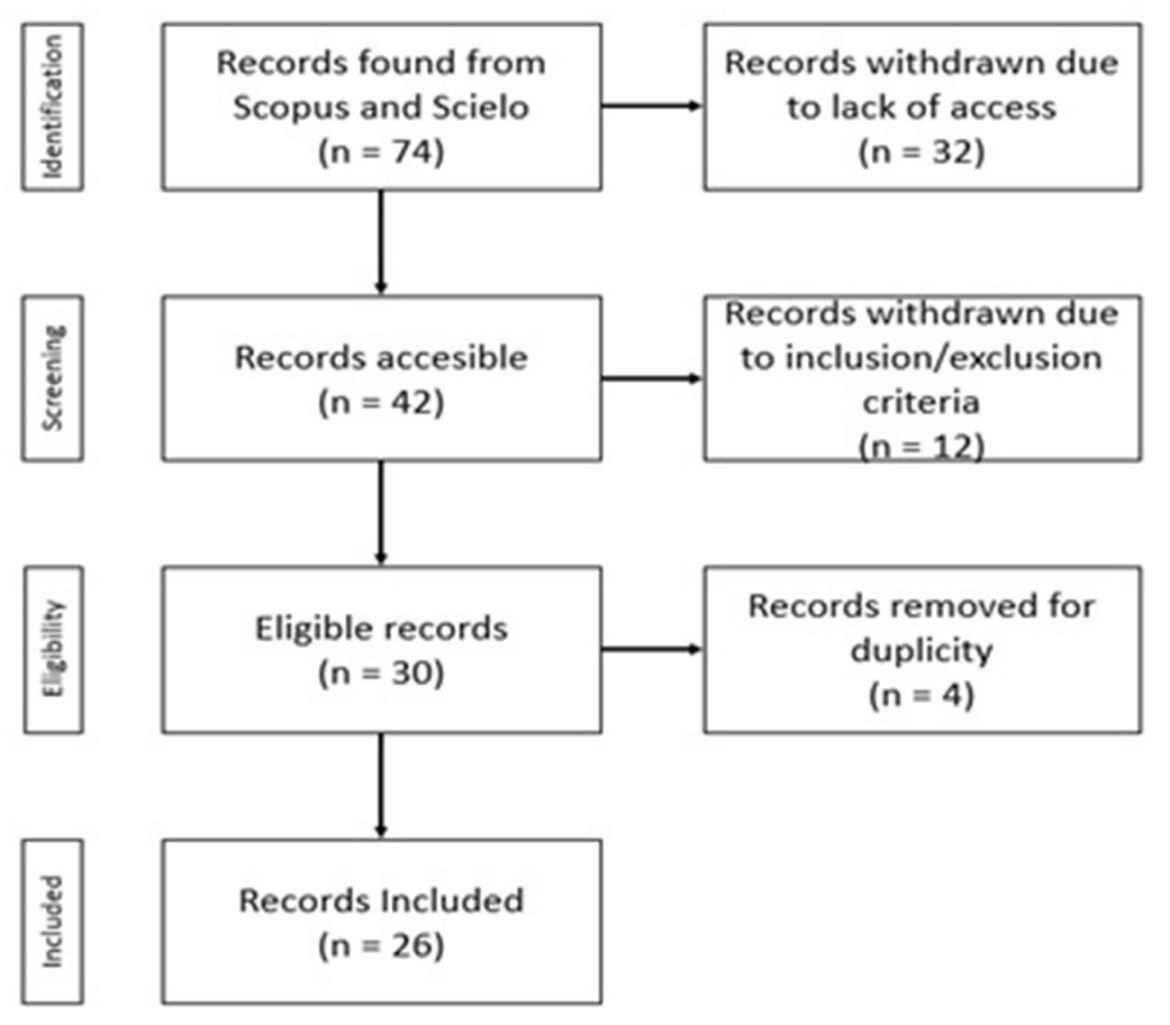
PRISMA flow chart.

To meet the objectives, the database was exhaustively reviewed and to categorize them, the titles, abstracts, and contents of each document were evaluated, extracting the data from the preselected and selected studies, to finally present the conclusions after analyzing and interpreting them. That said, the preferred reporting items for systematic review and meta-analyses (PRISMA) flow chart (36) was used for its presentation, which is detailed in Figure 1.

## Results

The 26 reorder identified were carefully reviewed and systematized and are shown in Table 2.

**Table 2:** Records identified.

**Code**	**Author**	**Tittle**	**Journal**
1	Oviedo, A. Delgado, I., and Licona, J. (2020) (37)	Habilidades sociales de comunicación en el cuidado humanizado de enfermería: Un diagnóstico para una intervención socioeducativa.	Escola Anna Nery
2	Cruz, C. (2020) (38)	La naturaleza del cuidado humanizado. Enfermería	Enfermería: Cuidados Humanizados
3	Díaz-Rodríguez, M., Alcántara, L., Aguilar, D., Puertas, E., and Cano, M. (2020) (39)	Orientaciones formativas para un cuidado humanizado en enfermería: una revisión integrativa de la literatura.	Enfermería Global
4	Silva, A., Pantoja, F., Millón, Y., Hidalgo, V., Stojanova, J., Arancibia, M., and Campos, M. (2020) (40)	Percepción de actores involucrados acerca del parto humanizado y la violencia obstétrica en Chile: una revisión panorámica.	Medwave
5	Correa-Pérez, L. and Chavarro, G. (2020) (41)	Integralidad en la atención del paciente crítico: buscando un camino para humanizar la UCI.	Acta Colombiana de Cuidado Intensivo.
6	Martínez, P., Suárez, N., Gómez, L., Bolívar, Y., and Rodríguez, É. (42)	Percepciones de dignidad y respeto en Unidades de Cuidado Intensivo.	Acta Colombiana de Cuidado Intensivo.
7	Ospina, D., Cristancho, S., Lafaurie, M., and Rubio, D. (2020) (43)	Humanización de los servicios reproductivos desde las experiencias de las mujeres: aportes para la reflexión.	Revista Cuidarte
8	Monje P., Miranda P., Oyarzün J., Seguel F., and Flores E. (2020) (44)	Percepción de cuidado humanizado de enfermería desde la perspectiva de usuarios hospitalizados	Ciencia Enfermería.
9	Campiño-Valederrama S., Duque P., and Cardozo-Arias V. (2020) (45)	Percepción del paciente hospitalizado sobre el cuidado brindado por estudiantes de enfermería.	Universidad y Salud.
10	Escobar-Castellanos B. and Cid-Henriquez P. (2018) (46)	El cuidado de enfermería y la ética derivados del avance tecnológico en salud.	Acta bioethica.
11	Yáñez-Dabdoub M and Vargas-Celis I. (2018) (47)	Cuidado humanizado en pacientes con limitación del esfuerzo terapéutico en cuidados intensivos: desafíos para enfermería.	Persona y Bioética.
12	Joven Z. (2019) (48)	Percepción del paciente crítico sobre los comportamientos de cuidado humanizado de enfermería.	Avances de Enfermería
13	Hernández L., Díaz A., Martínez J., and Gaytán D. (2018) (49)	Educación de enfermería en el cuidado humanizado.	Escola Anna Nery
14	Acosta-Romo, M., Cabrera-Bravo, N., Basante-Castro, Y., and Jurado, D. (2017) (50)	Sentimientos que experimentan los padres en el difícil camino de la hospitalización de sus hijos prematuros. Un aporte al cuidado humanizado.	Universidad y Salud
15	Beltran-Salazar, Ó. (2015) (51)	Atención al detalle, un requisito para el cuidado humanizado.	Index Enferm
16	Lopera B. (2016) (52)	Cuidado humanizado de enfermería al final de la vida: el proceso humanizado de muerte.	Revista Colombiana Enfermería
17	Mejía M., Faneyra F., Molina B., and Arango U. (2018) (53)	La deshumanización en el parto: significados y vivencias de las mujeres asistidas en la red pública de Medellín.	Investigación educación enfermería
18	Ramírez P. (2016) (54)	Fenomenología hermenéutica y sus implicaciones en enfermería.	Index Enfermería
19	Rojas V. (2019) (55)	Humanización de los cuidados intensivos humanization of intensive care.	Revista Médica Clínica las Condes.
20	García-Salido, A., La Calle, G. H., and González, A. S. (2019) (56)	Revisión narrativa sobre humanización en cuidados intensivos pediátricos: ¿‘dónde estamos?	Medicina Intensiva
21	Vialart N., Medina I., and Gavilondo X. 2018 (57)	La cultura profesional del docente de enfermería: Preparación ante las tecnologías informáticas.	Revista Cubana Enfermería.
22	Camero, Y., Meléndez, I., Álvarez, A., and Apuntes, Y. (2019) (58)	Cuidado Humanizado en el Postoperatorio Inmediato de Pacientes Histerectomizadas.	Cultura de los Cuidados.
23	Borges L., Sixto A., and Sánchez R. (2018) (59)	Comprehensive Perspective about Humanized Care to Women during Labor and Delivery.	Revista Cubana Enfermería
24	Castañeda C., Orozco M., and Rincón G. (2015) (60)	“Empoderamiento,” una utopía posible para reconstruir la humanización en Unidades de Cuidado Crítico.	Hacia promoción salud.
25	Pabón-Ortiz E., Mora-Cruz J., Buitrago-Buitrago C., and Castiblanco-Montañez R. (2021) (61)	Estrategias para fortalecer la humanización de los servicios en salud en urgencias	Revista ciencia cuidad.
26	Valenzuela, M., Sanjuan-Qui, Á., Ríos-Risquez, M., Valenzuela-Anguita, M. Juliá-Sanchis, and R. Montejano-Lozoya, R. (2019) (62)	Humanización de la asistencia en urgencias: un análisis cualitativo basado en las experiencias de las enfermeras.	Revista de Enfermagem Referência.

A more detailed analysis by the authors identified relevant data for each of the records, such as the country where the study was carried out, the methodology applied, the results, and the conclusions reached. This is detailed in Table 3.

**Table 3:** Details of the studies.

**Code**	**Country**	**Methodology**	**Results and conclusions**
1	Mexico	Quantitative, descriptive and cross-sectional study.	In relation to empathy and communication, patients perceived humanized care in the majority of nursing professionals at a high level. When evaluating respect and kindness, they wish to be understood and cared for. It is concluded that nursing professionals need to strengthen social skills and effective communication.
2	Chile	Literature review	During the processes of care, patients perceive a vertical—unidirectional relationship of professional-patient. The categories for the practice of humanized care are: the human being, professional-patient relationship, subject of care, communication and holistic approach.
3	Spain	Integrative bibliographic review	The need is expressed to incorporate training programs in emotional and communication competencies in nursing in order to strengthen humanized care.
4	Chile	Systematic review	The analysis carried out by nursing professionals identifies areas of conflict and consensus, as diverse interacting dimensions that should be addressed with the development of comprehensive and effective health policies, because they hinder the advancement of hospital humanization.
5	Colombia	Literature review	In the healthcare field, there are efforts to raise the psychoemotional aspects directed to the patient—family, of the intensive care areas. It is concluded that the ABCDEF care rates are a way to humanize the care of the patient in critical condition.
6	Colombia	Mixed quantitative-qualitative approach.	It is concluded that the technified language leads to a distance between patient-health personnel, since technology dehumanizes the work of health personnel, the doctor-patient relationship, now: doctor-machine-patient, where the machine seems to legitimize the patient's discourse.
7	Colombia	Qualitative approach.	The factors related to the lack of humanization are: lack of training, lack of competencies, compassion, mindfulness and soft skills; added to the lack of information in the area of specialization and the increase of the nurse-patient ratio. It is concluded that the strategies improve empathy, communication and skills for successful care, reduce work overload in the area.
8	Chile	Quantitative approach, correlational study, with a sample of 171 hospitalized patients.	Humanized care exhibits categories in doing, identifies the patient's needs, maintains a cordial treatment, availability and educates. It is concluded that nursing professionals in a high complexity hospital respect human dignity and provide daily humanized care.
9	Colombia	Quantitative, descriptive, cross-sectional, quantitative approach study, conducted with 356 patients.	Patients perceive humanized care in the attention, highlighting the identification of physical, psychological and spiritual needs that achieved low scores. It is concluded that humanized care is a phenomenon of interest for the discipline, by identifying strengths and weaknesses in care.
10	Chile	Literature review	The nursing professional should demonstrate humanized care in all the actions he/she performs, applying ethical knowledge, attitudes and values to the person to be cared for.
11	Chile	Qualitative approach study.	It is concluded that in humanized care, the roles of nurses as defenders of the patient's interests and communicating agents stand out.
12	Colombia	Quantitative, descriptive, descriptive approach study. It was applied to 55 patients admitted to the ICU.	It is concluded that the actions of humanized care are globally perceived as good, in the category Prioritizing the subject of care for patient satisfaction.
13	Mexico	Pre-experimental, quantitative approach study with 37 nurses.	It is concluded that the educational intervention increased the knowledge on the good use of the dignified treatment indicator.
14	Colombia	Qualitative approach study with eight parents between 17 and 35 years of age.	The results show that feelings and affective bonding are expressions of parental love and of the process of interaction with health personnel.
15	Colombia	Qualitative, phenomenological approach study with 16 participants.	It is concluded that humanized care includes details that favor nurse-patient interaction.
16	Colombia	Qualitative, ethnographic approach study.	The results show the attributes of a humanized death as a dignified death: the person keeps his/her rights; calm death: when he/she keeps his/her preferences; good or peaceful death: when it is accepted; and beautiful death: when death occurs at home. It is concluded that humanized death requires the participation and leadership of the nurse with caring actions.
17	Colombia	Qualitative approach study	The results show that the meaning of the experiences of the birthing process are not consistent with a transcendent human experience.
18	Colombia	Qualitative approach study	It is concluded that individualized care from the perspective of the unitary being humanizes the practice of care.
19	Chile	Literature review	The technological progress achieved in the diagnosis and treatment of diseases has not gone hand in hand with advances in the development of non-technical skills in the health team, the latter demanded and suffered by patients and relatives, who yearn for comprehensive care, in a context of crisis in ICU hospitalization.
20	España	Literature review	Humanization should be the subject of debate, without calling into question the humanity displayed by professionals. In this study, the strategic lines on which the humanized care of the critically ill patient revolves were analyzed and referred to, adapting them to the pediatric setting.
21	Cuba	Bibliographic and documentary review	Informatics should be seen as an instrument that facilitates the management of humanized care from the interaction of professionals and care actors. Its application should be valued to provide care and promote health with quality and ethics, which does not exclude its realization with humanity and respect.
22	Ecuador	Quantitative, descriptive approach.	Humanized care is a complex and indispensable process for post-operative care in hysterectomies. In the health institution approached, the care is developed in a fragmented way, since there is an opening in the nursing professionals for humanization.
23	Cuba	Systematic literature review	The relevance of the humanized care provided to women during labor is based on the fact that it leads to the satisfaction of physical, emotional and spiritual needs.
24	Colombia	Mixed study Integrative review of scientific and qualitative literature: descriptive-phenomenological.	Empowerment as a disciplinary tool allows facing the difficulties of professional role identity and adherence to Watson's Theory of Human Care, in juxtaposition with the Nursing Diagnosis Taxonomy-NANDA, as a proposal that allows transcending the barriers of care in Critical Care Units.
25	Colombia	Integrative review	The strategies identified as effective and that have an impact on low humanization are: lack of training, competence and soft skills, compassion and mindfulness; added to the lack of information in expert areas and a decrease in the nurse-patient relationship. It is concluded that the strategies mentioned improve empathy, raise awareness of the present, favor communication and facilitate practices for the success of care and reduce work overload.
26	Spain	Qualitative approach study	Strengthen initiatives to implement integrated health care models. The implementation of holistic, patient- and family-centered care is essential to ensure the humanization of health care in the emergency department.

## Discussion

Articles pertaining to humanized care from the point of view of nurses–patients in a hospital context were found in the two databases. The articles were selected according to the objectives of the search, obtaining a total of 26 articles from six countries (Spain, Chile, Mexico, Cuba, Ecuador, and Colombia), which were quantitative (6), qualitative (9), mixed (1), and systematic review (10), which were critically analyzed to meet the objectives of the study.

Humanized nursing care is a unique way of caring within the health system, where the patient is offered a safe environment and protection of their human dignity, which sustains the care over time, educates to promote health and harmony in body, mind, and soul, based on a relationship of trust. Likewise, humanized nursing care attends to the human and therapeutic needs of the patient in his or her health–disease condition (13, 54).

The evidence suggests that from the perception of nurses–patients, the topic leads to discussing and reflecting on the complexity that humanized care implies, from dignified treatment to the application of institutional policies, for which the commitment of all social actors is required to assist in humanizing health institutions. Expressions that are perceived by nurse–patients in the hospital context are detailed.

### Elimination of the Constraints of the Delivery of Humanized Care

Prieto Martínez et al. (42) state that patients and family members perceive limitations in humanized care related to the technified language that causes distance and distrust, and in health personnel they perceive a greater interest in technological equipment, diagnoses, and disease evolution, which brings with it a feeling of disinterest in the person and a lack of compassion in front of the suffering, and pain experienced by the person and the family.

Also, Ospina Vanegas et al. (43) point out that health professionals show a lack of soft and social skills, which makes it difficult for them to achieve adequate interpersonal relationships, especially in critical situations experienced by patients (21, 24). In fact, the health problems faced by patients lead to strict changes in their lifestyle, which generates emotional and even existential crises, with some physical limitations to carrying out their daily activities.

In this sense, health professionals assume critical behavior due to the same health situation experienced by patients, and the strict pressures to comply with medical indications generates insecurity, fear, and apprehension, resulting in distancing for interpersonal relationships and even little freedom to express doubts about emerging health scenarios, turning them into simple listeners, which hinders the achievement of joint goals designed by the nursing staff.

In relation to the infrastructure of health services, in some cases these do not meet the needs of patients as they do not protect privacy, do not provide comfort, and there is no waiting room for family members, among others. In addition, the lack of institutional policies does not allow promoting behavioral change of professionals within health institutions, who continue to label the patient with a bed number, a diagnosis, name of the disease, among others; who currently demand health services based on the right, with the freedom to make complaints or complaints in the institutions for mistreatment, misinformation, lack of medicines or disinterest in the health situation, among others.

Cruz C. argues that the hegemony of the medical paradigm wields a vertical-unidirectional relationship between the professional and the patient, where decision making is an obstacle to the joint work between health professionals–patient and family. However, the management of hospital services from a patient-centered vision favors the quality of health services with a sense of accessibility, social inclusion, and health for all from a humanizing vision (42). Likewise, Diaz Rodríguez et al. (39) report that deficiencies in professional training limit the competencies and soft skills of professionals, who are unable to deal with situations of emotional imbalance experienced by the patient during the care process.

In short, the various limitations in hospital systems affect management, block the expectations of patients and relatives, and the continuity of health care with the various phenomena experienced by the sick person and which are linked to suffering due to the disease itself and to treatment-care, because care is a service that requires faith, hope, and love. Considering that caring is an art and a science, it turns the service into a moment of transcendent gratitude.

### Humanizing Nursing Role in Hospital Services, Especially in Critical Areas

According to international studies, participation and leadership of nurses in care actions are critical to supporting quality standards because of their role in defending the rights of the patient and acting as communication agents of care (46). According to Oviedo et al. (37), humanizing behavior creates a calm and confident environment for the health scenario that is being experienced, an affirmation that can be validated when patients yearn within the care attention process to establish affective bonds of trust with details of cordiality, kind treatment, active listening, empathy, solidarity, and respect for customs and beliefs (37, 48, 51).

According to Silva et al. (40), nursing professionals have to deal with highly complex services, most of whom respect human dignity by providing humanized care on a daily basis. However, these critical areas necessitate a high level of specialization and emotional skills from professionals to respond to the needs of the patient and family who live in a state of uncertainty due to the same situation or health condition of the patient. So professionals must be trained to deal with such situations from the standpoint of ethical knowledge of care (40).

In this regard, it is necessary to implement and strengthen professional and technical competencies and soft skills in nurses of critical services: emergency, post-operative, intensive care unit, pediatrics, maternity, oncology, among others, to provide holistic care focusing on the physical, psychological, and spiritual dimensions, among others (41, 45, 55, 56).

On the other hand, in the case of maternity services, women express the various emotions they experience in adapting to a new way of life. It should be noted that the process of childbirth is important for a woman, but it requires humanized care, where the health personnel can identify her needs, offer her cordial treatment, be available, and educate her so that she can deal with her own self-care and in the management of the integral care of her child (53).

In relation to areas where there is a high mortality rate, health professionals should generally have the appropriate competencies to address bereavement situations and care for a dignified death (52). Likewise, Correa-Pérez and Chavarro (41) state that nurses should make great efforts to train themselves in addressing the psychoemotional aspects of patients so that they can achieve relationships of trust that allow them to raise the quality of the services provided by the nurse.

### Indicators That Favor the Implementation of Humanized Care

Humanized care includes the nurse–patient–family relationship, which strengthens the work performance and productivity levels of nurses who perform not only procedural activities, but also provide information and emotional, spiritual, and educational support, which raises the quality indicators in the care processes, safe environment, promotion of self-care, health education, and reduction of operating costs during the health care process, thus ensuring the sustainability of health care over time. Continuous training of care actors should promote the use of the dignified treatment indicator (49, 57).

### Strategy and Innovation for a Path of Humanization

It is necessary to identify and prioritize health needs to assume the practice of human care for patients, considering the different human dimensions: physical, psychological, spiritual, among others; with the purpose of maintaining a predisposition to provide continuous care, promote dignified and cordial treatment, and motivate a safe environment that favors the process of care based on values (45). Caring requires the development of skills and the meeting of knowledge because it requires the capacity for active listening, kind response, and constant training to achieve the path to humanization.

During the outcome of care practice, nurses adhere to the Jean Watson theory to transcend in their work and achieve a balance of theoretical and practical approach that generates confidence during the health care process. Strengthening the soft skills of health professionals enables them to adequately increase interpersonal relationships that will support the psychological emotional aspects of patients, as well as maintain education and ongoing training of the patient and family (39, 41, 50).

Finally, humanized care is a way of life that the nursing professional assumes in order to identify the needs, expectations, and health demands of patients and family members who require professional nursing care. As a result, it is critical that the patient perceives highly qualified personnel with whom he or she can maintain trust bonds throughout the health-disease process, taking into account the patient's disposition, commitment, cordiality, kindness, simple language, and availability of care. However, it is necessary that institutional policies are applied to generate spaces for analysis to achieve quality indicators, according to international standards that consider humanized care as an essential right in the hospital context (58–62).

## Conclusion

This systematic review evidences the knowledge gap regarding the development and/or construction of care models on humanized care in public and private institutions worldwide, a limitation that should become a worldwide challenge for the application of humanizing policies and models for practice and that should be linked to the professional practice of health personnel, especially nursing professionals, highlighting the prioritization of the basic needs of patients who should receive humanizing care that responds to social changes.

Since nursing professionals lack competencies and soft and social skills, patients are put in danger because they are not cared for in a comprehensive, quality, and warm manner.

It should be noted that care should reinforce the sense of trust to respond to their basic therapeutic needs based on an authentic safe care approach to the production of assistive care, which is an art that requires a willingness to care and share feelings and emotions during the interpersonal and transpersonal relationship between the caregiver and the patient.

## Data Availability Statement

The systematic review was carried out using scientific articles available in the open access databases Redalyc and Scielo.

## Author Contributions

All authors listed have equally contributed to the work and approved it for publication.

## Funding

This study was carried out and funded by the Universidad César Vallejo, within the framework of the work plan outlined in RVI N° 052-2019-VI-UCV.

## Conflict of Interest

The authors declare that the research was conducted in the absence of any commercial or financial relationships that could be construed as a potential conflict of interest.

## Publisher's Note

All claims expressed in this article are solely those of the authors and do not necessarily represent those of their affiliated organizations, or those of the publisher, the editors and the reviewers. Any product that may be evaluated in this article, or claim that may be made by its manufacturer, is not guaranteed or endorsed by the publisher.
